# Unveiling a novel *GJB2* dominant K22T mutation in a Chinese family with hearing loss: A novel GJB2-dominant K22T mutation

**DOI:** 10.3724/abbs.2024064

**Published:** 2024-05-10

**Authors:** Haiting Ji, Yilai Shu, Huawei Li

**Affiliations:** 1 Department of the Affiliated Eye and ENT Hospital State Key Laboratory of Medical Neurobiology ENT Institute and Otorhinolaryngology Fudan University Shanghai 200031 China; 2 NHC Key Laboratory of Hearing Medicine Fudan University Shanghai 200031 China; 3 Shanghai Engineering Research Centre of Cochlear Implant Shanghai 200031 China; 4 ENT Institute and Otorhinolaryngology Department of Eye & ENT Hospital State Key Laboratory of Medical Neurobiology and MOE Frontiers Center for Brain Science Fudan University Shanghai 200031 China; 5 Institutes of Biomedical Sciences Fudan University Shanghai 200032 China

**Keywords:** deafness, point mutations, *GJB2*, next-generation sequencing

## Abstract

Hearing loss constitutes one of the most prevalent conditions within the field of otolaryngology. Recent investigations have revealed that mutations in deafness-associated genes, including point mutations and variations in DNA sequences, can cause hearing impairments. With the ethology of deafness remaining unclear for a substantial portion of the affected population, further screenings for pathogenic mutations are imperative to unveil the underlying mechanisms. On this study, by using next-generation sequencing, we examine 129 commonly implicated deafness-related genes in a Chinese family with hearing loss, revealing a novel heterozygous dominant mutation in the *GJB2* gene (GJB2: c.65T>G: p. Lys22Thr). This mutation consistently occurs in affected family members but is not detected in unaffected individuals, strongly suggesting its causative role in hearing loss. Structural analysis indicates potential disruption to the Cx26 gap junction channel’s hydrogen bond and electrostatic interactions, aligning with predictions from the PolyPhen and SIFT algorithms. In conclusion, our study provides conclusive evidence that the identified heterozygous *GJB2* mutation (GJB2: c.65T>G: p. Lys22Thr), specifically the K22T alteration, is the primary determinant of the family’s deafness. This contribution enhances our understanding of the interplay between common deafness-associated genes and hearing loss, offering valuable insights for diagnostic guidance and the formulation of therapeutic strategies for this condition.

## Introduction

Hearing loss, a prevalent sensory impairment, affects a substantial portion of the global populace, with an estimated 5% of individuals worldwide experiencing this condition [1]. Among these, congenital or prelingual deafness impacts approximately 1 in 1000 children [ 2‒ 4]. The ramifications of hearing loss are profound, encompassing challenges in language acquisition, hindered educational attainment, and the potential for psychological distress, social isolation, and reduced quality of life (QOL) [ 1, 5, 6]. Consequently, elucidating the intricate mechanisms governing hearing loss holds great promise for advancing therapeutic strategies.

The etiology of hearing loss arises from a complex interplay of factors, including genetic predisposition, aging, pharmaceutical influences, and viral infections [ 4, 7]. Notably, genetic factors account for approximately 60% of congenital sensorineural hearing loss cases [ 8, 9]. In recent years, a comprehensive repertoire of genes associated with deafness has emerged, with point mutations within these genes identified as causative agents in various hearing loss cases [10]. Noteworthy examples include mutations in the *GJB2* gene encoding connexin26 (Cx26), which is responsible for more than half of autosomal recessive non-syndromic hearing loss cases [ 8, 11‒ 13]. Additionally, mutations in *GJB4* (Cx30.3), including frameshift mutations and amino acid variants such as R103C, R124Q, R160C, C169W, and E204A, have been linked to non-syndromic hearing loss [ 14, 15]. Moreover, genes such as *USH2A*, *USH2C*, and *USH2D* have been implicated in Usher syndrome, a hereditary disorder characterized by combined auditory and visual impairment [ 16, 17]. Further expanding the genetic spectrum, mutations in the *KCNQ1* gene have been associated with Jervell and Lange-Nielsen (JNL) syndrome, which features congenital deafness alongside prolonged QT intervals in electrocardiograms [ 17, 18]. Despite these advancements, a significant proportion of affected individuals still lack a clear etiological understanding of their deafness, underscoring the need to elucidate the intricate relationship between hearing loss and deafness-associated genes. Thus, a systematic identification of pathogenic mutations remains pivotal, which will shed light on the mechanistic underpinnings of deafness and offer prospects for targeted therapeutic interventions [ 19, 20].

Distinct from the predominant autosomal recessive pattern associated with most *GJB2* point mutations, instances of autosomal dominant non-syndromic hearing loss have been well documented [21]. Notably, individuals who experience both hearing impairment and skin disorders often exhibit a dominant pattern of syndromic inheritance. In contrast, our investigation revealed a novel heterozygous mutation linked specifically to non-syndromic phenotypes, notably without any occurrence of skin-related conditions among the family members. This distinction is of paramount importance, considering that non-syndromic deafness primarily arises from loss-of-function mutations, while the literature underscores a gain-of-function mechanism in syndromic deafness patients [22].

Given the intricate genetic landscape contributing to hearing loss, the adoption of next-generation sequencing (NGS) has emerged as an indispensable strategy for unveiling novel gene mutations [23]. In line with this paradigm, our study harnesses the well-established NGS approach to unravel previously unrecognized disease-causing mutations within a Chinese family with non-syndromic hearing loss.

## Materials and Methods

### Family enrollment

The study cohort included a proband (IV-1), a 6-year-old male exhibiting delayed speech development, who received a diagnosis of severe-to-profound sensorineural hearing loss at the Department of Otolaryngology, EENT Hospital of Fudan University (Shanghai, China). Overall, the entire family was enlisted from participation, resulting in the recruitment of 12 individuals. All participants were provided with a comprehensive understanding of the study’s scope and requirements, and their informed consent was secured through the formal signing of patient consent forms, which received ethical approval from the Fudan University Ethics Committee. The study was conducted in accordance with the Declaration of Helsinki and approved by the Ethics Committee of the EENT Hospital of Fudan University (Approval number: 2,017,044.) for studies involving humans.

### Clinical evaluation

Across three generations, all affected family members were clinically diagnosed with sensorineural hearing loss. Meticulously conducted histories focused on aspects such as hearing impairment, pregnancy and labor experiences, general health status, and chronic health conditions. These assessments were pivotal for excluding potential alternative causes of hearing loss, including pre- or postnatal risk factors such as neonatal complications, bacterial meningitis, infections, the use of ototoxic medications, and instances of head trauma. Audiological evaluations unveiled bilateral, symmetrical, severe-to-profound sensorineural hearing loss with a characteristic sloping configuration. Comprehensive otoscopic examinations and thorough physical evaluations were administered to all family members. Remarkably, these evaluations collectively highlighted bilateral, severe-to-profound sensorineural hearing loss, which exhibited either prelingual or postlingual onset. Furthermore, ancillary assessments, including funduscopic examinations, tandem gait tests, and Romberg tests, were performed to assess visual impairment and vestibular function [24]. Notably, no other salient clinical findings were evident within this familial context.

### DNA extraction and next-generation sequencing

Adhering to established protocols [25], genomic DNA was extracted from blood samples (3 to 5 ml) collected from both affected and unaffected family members. The QIAamp DNA Blood Maxi Kit (Qiagen, Valencia, USA) facilitated this extraction process, followed by stringent quality assessment, which included optical density ratio (260/280 ratio) measurements and gel electrophoresis imaging. To validate the mutations identified via NGS, PCR-based Sanger sequencing was meticulously performed by an external commercial entity (MacroGen, Rockville, USA). Additionally, the assessment of mutations within *GJB2* and *GJB4* involved the application of the conventional Sanger sequencing technique, which was performed in collaboration with a commercial source (Beckman Coulter Genomics, Danvers, USA).

### Homology modeling and molecular dynamics simulations

The Protein Data Bank is the source of atomic coordinates for Gap-junction protein beta 2 (PDB code: 2zw3). With Gap-junction protein beta 2 serving as a template due to its commendable sequence similarity (~71.9%), the creation of a homologous structure for Gap-junction protein beta 4 was realized via the SWISS-MODEL server. To achieve a balanced structure, a series of molecular dynamics simulations were thoughtfully conducted. To further expand our investigations, the structures of Cx26-K22T and Cx30.3-E204A were generated utilizing the “mutate” module embedded within Discovery Studio (Accelrys, San Diego, USA).

## Results

### Family and clinical presentation

**Figure 1:**
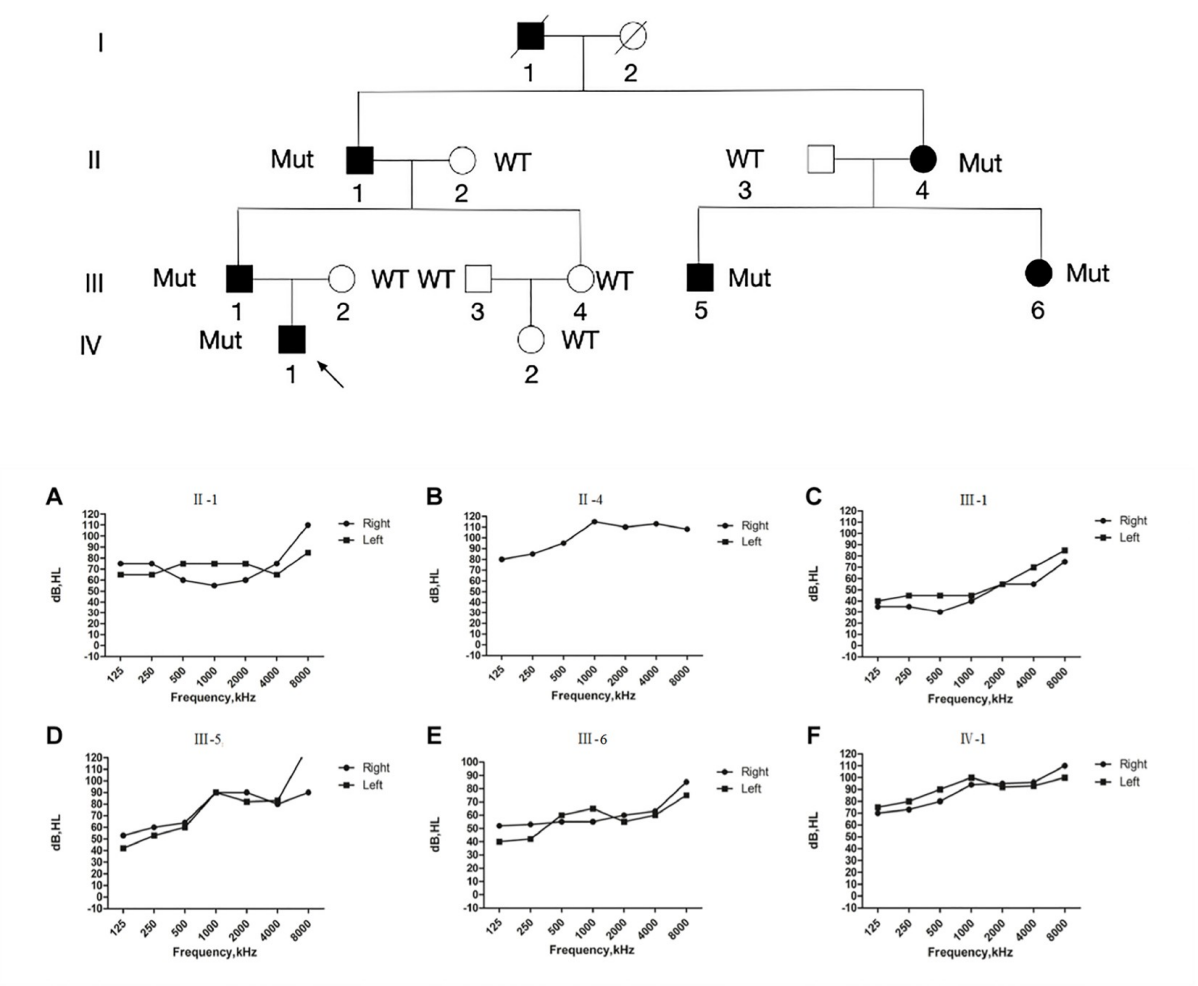
Figure 1 Pedigree and PTA of the Chinese family The circle symbols represent women and squares indicate men. Open symbols mean unaffected patients and close symbols mean affected patients. The proband is indicated by an arrow and deceased persons are indicated by a diagonal line through the symbol. (A–F) The PTA was detected in affected members in the Chinese family. Frequency in hertz (Hz) is plotted on the x-axis and the auditory threshold in decibels (dB) on the y-axis. Round, right ear; square, left ear.

Our investigation included the participation of individuals from a multigenerational Chinese family, including six affected patients (II-1, II-4, III-1, III-5, III-6, and IV-1), as well as six unaffected family members (II-2, II-3, III-2, III-3, III-4, and IV-2), as illustrated in Figure 1. A comprehensive evaluation protocol, consisting of pure-tone and acoustic immittance testing, otoscopic examinations, and comprehensive physical assessments, was conducted for both the affected and unaffected individuals. Moreover, the affected subjects underwent supplementary assessments, including funduscopic, tandem gait, and Romberg tests, to detect any manifestations of vision impairment or vestibular dysfunction. The audiometric assessments revealed characteristic bilateral, symmetrical severe-to-profound sensorineural hearing loss with a distinctive sloping pattern. The pure-tone audiometry (PTA) results, depicted in Figure 1, indicated that hearing loss ranged from mild to severe in six affected patients. The absence of vestibular, visual, or dermatological abnormalities in the affected individuals substantiated the diagnosis of non-syndromic hearing loss ( Table 1).

**Table:** **Table 1** Clinical manifestations of the patients with deafness

Generation	2 ^nd^	3 ^rd^	4 ^th^
Ⅱ-1	Ⅱ-4	Ⅲ-1	Ⅲ-5	Ⅲ-6	Ⅳ-1
Gender	Male	Female	Male	Male	Female	Male
Consult age	63	57	35	36	33	7
Onset age	18	4	12	10	1	2
Deafness	Yes	Yes	Yes	Yes	Yes	Yes
Prelingual	Yes	Yes	Yes	Yes		
Postlingual					Yes	Yes
Vision loss	None	None	None	None	None	None
Vestibular dysfunction	None	None	None	None	None	None
Skin disorders	None	None	None	None	None	None

### Identification of candidate mutations

Our endeavor to identify the disease-causing mutation within the Chinese family encompassed targeted capture of deafness-related genes, followed by next-generation sequencing. Among the array of mutations detected, a novel *GJB2* missense mutation ( *GJB2*: c.65T>G: p. Lys22Thr) emerged as a previously unreported variant, exhibiting a high probability of being pathogenic, as predicted by both the SIFT and PolyPhen2 algorithms. Interestingly, a heterozygous *GJB4* mutation ( *GJB4*c. 611A>C: p. Glu204Ala) was also discerned; however, its significant population allele frequency (PAF) within the 1000 Genomes Project data suggested a lesser role as the primary causative factor for deafness. Intriguingly, although *GJB4*c. 611A>C: p. Glu204Ala) displayed a global minor allele frequency (MAF) of 0.108, classifying it as a high PAF mutation ( Table 2). Analyses conducted through SIFT and PolyPhen indicated the potential of this *GJB4* mutation to disrupt the encoded connexin 30.3 protein, thereby suggesting its contribution to the risk of deafness ( Table 2). These findings collectively implicate *GJB2* mutation as the foremost candidate for hearing loss ethology while suggesting an intriguing association between *GJB4* mutation and hearing loss within this familial context.

**Table:** **Table 2** The common mutations of all affected family numbers

Gene name	Point mutation	Details of point mutations	Mutation type ^1^	Global MAF	SIFT	Poly-Phen	Category
*GJB2*	Chr13: 20763656 T>G	NM_004004:exon2: c. A65C: p. K22T	Het	-	0 ^2^	0.999 ^1^	VI
*GJB4*	Chr1: 35227466 A>C	NM_153212:exon2: c. A611C: p. E204A	Het	0.108 (C)	0	1	high PAF ^3^

^1^Mutation type: het, heterozygous; hom, homozygous. ^2^The SIFT and Poly-Phen analysis were performed using online service. Poly-Phen ( http://genetics.bwh.harvard.edu/) and SIFT ( http://sift.jcvi.org/). ^3^Since the globe MAF (Minor Allele Frequency) of *GJB4* (Chr1: 35227466 A>C) is 0.108, which is a bit higher than the grouping standard, we identify this mutation as high PAF mutation. However, the SIFT score is 0 and Poly-Phen is 1. Thus, this kind of mutation is likely to induce damage of the protein of connexin30.3 and may also contribute to deafness.

### Validation and structural analysis

**Figure 2:**
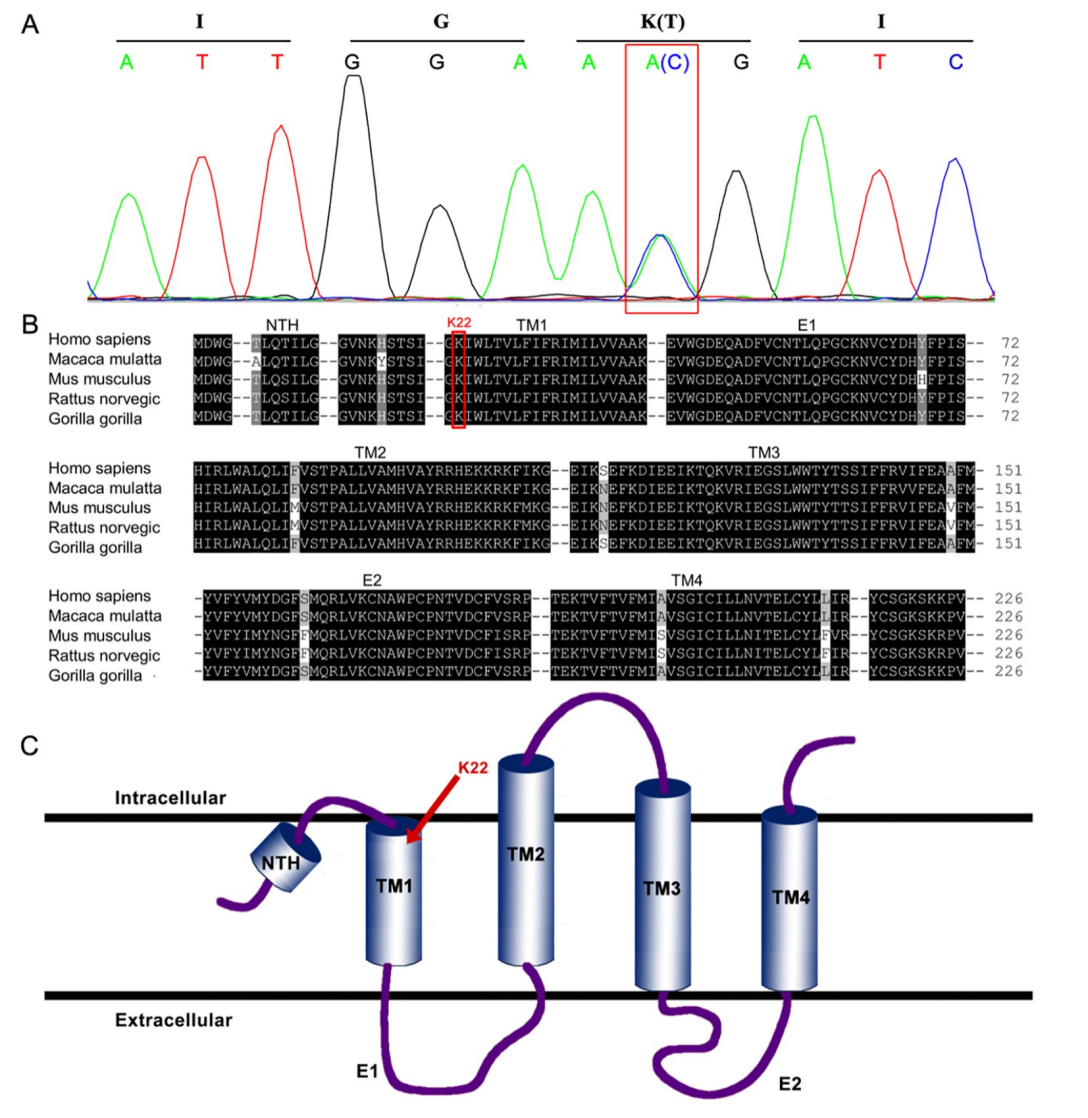
Figure 2 Mutation validation, conservation analysis and protomer structure of GJB2 (A) The identified mutation was confirmed by Sanger sequencing. The boxes indicate the location of the nucleotide changes. (B) Sequence alignment showing the Lys22 is a conserved residue among different species. NTH, N-terminal helix; TM1~TM4, transmembrane helices1~4; E1~E2, extracellular loops 1~2. (C) Topological diagram of the Cx26. The mutated site is indicated by arrow.

Validation of the novel *GJB2* mutation was performed through meticulous Sanger sequencing ( Figure 2A). Sequence analysis pinpointed the location of the mutation within the intracellular segment of transmembrane domain 1 (TM 1), further accentuating its conservation across diverse species. This finding underscores the indispensable role of Lys22 in preserving the functionality of Cx26 ( Figure 2B,C). These combined results reinforced the pivotal involvement of the *GJB2* mutation in precipitating hearing loss within this familial context.

### Structural insights

**Figure 3:**
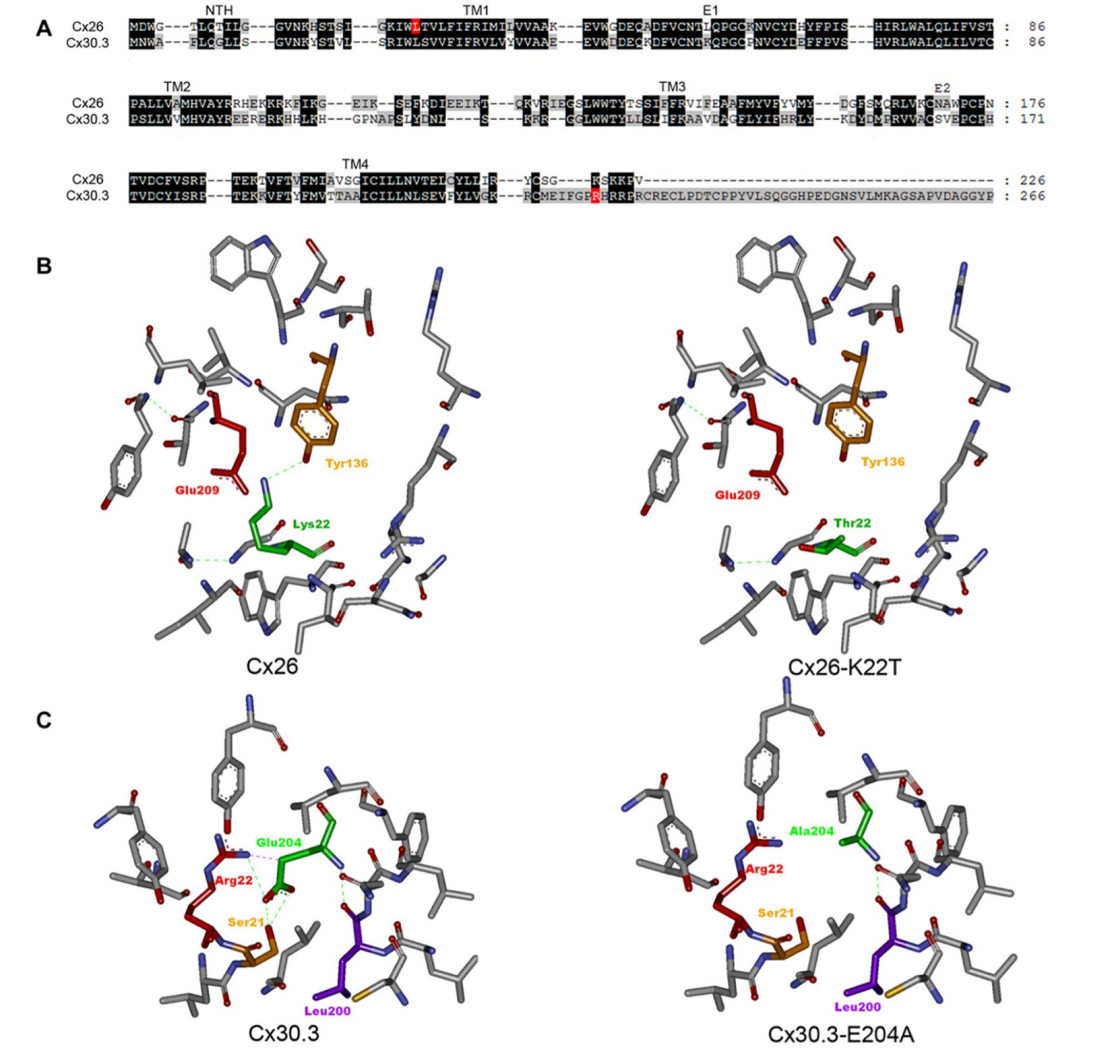
Figure 3 Structure views of Cx26, Cx30.3 and their mutations (A) Sequence alignment of Cx26 and Cx30. The sequence similarity is 71.9% while the sequence identity is 51.1%. (B) Structure views of Cx26 (left) and mutant Cx26-K22T (right). The mutant structure was constructed using Discover Studio. Main residues surrounding Lys22 within 10 Å, in grey; hydrogen bonds, green dotted lines. The H-bond between Tyr136 and Lys22 disappears when Lys22 is mutated into Thr22. The substitution of lysine with threonine also disrupts the possible electrostatic interaction between Lys22 and the neighbor residue Glu209. (C) Structure views of Cx30.3 (left) and Cx30.3-E204A (right). The homologous structure of Cx30.3 was constructed using SWISS-MODEL server. Further mutation was performed in Discover Studio. Main residues surrounding Glu204 within 10 Å. The H-bonds between Glu204 and the neighbor residues Ser21, Arg22 and Leu 200 are absent in E204A. The substitution of glutamate with alanine also disrupts the electrostatic interaction between Glu204 and the neighbor residue Arg22.

Gap junction proteins, as encoded by GJB genes, represent a family of transmembrane proteins facilitating intercellular communication channels. In recent years, the identification of an array of mutations in Cx26 and Cx30.3 within the deafness-affected population has underscored the intimate connection between gap junction proteins and hearing loss [ 8, 12, 25]. Employing homology modeling and molecular dynamics simulations, we embarked on a journey to decipher the structural implications of disease-causing mutations. Recognizing the enigmatic highMAF of the GJB4 mutation (approaching the threshold) and its potential to perturb the encoded protein (as illuminated by PolyPhen and SIFT predictions), we extended our analysis to Cx30.3. Drawing upon the robust sequence identity shared between Cx26 and Cx30.3 ( Figure 3), we harnessed the SWISS-MODEL server to construct the homologous structure of Cx30.3, employing the atomic structure of Cx26 as a scaffold. Subsequent molecular dynamics simulations were performed to attain a stabilized structure. To further illuminate the structural consequences, we introduced mutations in Cx26 and Cx30.3 through the use of Discover Studio software. As shown in Figure 3B, the substitution of Lys22 with threonine not only disrupted the formation of hydrogen bonds between Lys22 and Tyr136 but also obliterated the strong electrostatic interactions with the neighboring acidic residue Glu209. Given the strategic positioning of Lys22 within the pore-lining TM1 segment, we postulated that this mutation could induce a conformational shift in Cx26. Importantly, our sequence alignment revealed the correspondence of Glu204 in the Cx30.3 protein with Glu209 in Cx26. In the context of Cx30.3, Glu204 was unfolded by an array of nonpolar and polar residues (Ser21, Arg22, Tyr131, Leu135, Leu200, Asn201, Leu202, and Phe206), each within a contact distance of 5 Å. The substitution of Glu204 with the shorter side-chain nonpolar alanine resulted in the dissolution of hydrogen bonds and electrostatic interactions, culminating in structural perturbations ( Figure 3C). Through these intricate structural analyses, we gleaned insights indicating the potential for both *GJB2* and *GJB4* mutations to trigger modifications in the structure of gap junction proteins.

## Discussion

In this study, we undertook a comprehensive investigation encompassing clinical, genetic, and molecular aspects within a Chinese family with deafness. Leveraging a dual approach of Sanger sequencing and next-generation sequencing, we successfully unveiled novel mutations within genes pertinent to hearing impairment. Notably, a *GJB2* mutation ( *GJB2*: c.65T>G: p. Lys22Thr) emerged as a plausible genetic determinant of the observed deafness within this familial context.

Gap junction channels, which are distributed ubiquitously throughout human tissues, are pivotal conduits that facilitate intercellular signal transmission. Their profound influence extends across a spectrum of physiological and pathological processes [26]. Crucially, these channels orchestrate the movement of potassium ions within hair cells, thereby exerting pronounced control over the functional dynamics of the inner ear. At the structural level, the gap junction channel comprises two hemichannels, each composed of six connexin subunits. This diverse connexin family, encompassing more than 20 distinct members characterized by molecular weights, converges on a shared 3D structural framework despite variations in their expression patterns and physiological roles [ 27, 28]. Each connexin subunit is composed of 4 transmembrane segments (TM1~4), 2 extracellular loops (E1 and E2), a cytoplasmic loop, a C-terminal segment, and an N-terminal helix (NTH). The transmembrane domains, particularly TM1 and TM2, significantly contribute to pore formation by shaping the central architecture, whereas TM3 and TM4 are directed toward the hydrophobic lipid environment, shielding the gap junction against electrostatic influences from the surrounding membrane [13].

In our current investigation, we successfully diagnosed a novel *GJB2* mutation ( *GJB2*: c.65T>G: p. Lys22Thr) within the affected family. Through rigorous sequence alignment analyses, we ascertained the conservation of the mutated residue, Lys22, across diverse species ( Figure 2), particularly within the pore-lining helix TM1. This position assumes critical importance due to its proximity to other structural elements, including the hydrogen-bonding partner Tyr136 in TM3, as well as the neighboring acidic residue Glu209, which is separated by a mere 5 Å. This spatial arrangement implies at a robust electrostatic interaction. Using the crystal structure of Cx26 (PDB code: 2zw3) as a template, we constructed a structural model of the Cx26-K22T mutant using homology modeling and molecular dynamics simulations. Structural analysis revealed that the substitution of Lys22 with an electrically neutral threonine disrupts both the electrostatic interaction and the hydrogen bond network, ultimately inducing a conformational transition in connexin26 ( Figure 3B). Given that *GJB2 is* predominantly expressed in the inner ear, particularly the cochlea, we propose that the newly identified *GJB2* mutation contributes to the onset of hearing loss through its disruptive effect on the structural integrity of gap junction channels.

Redirecting our focus to *GJB4*, the gene encoding connexin 30.3, we recognize its significance as a genetic risk factor for nonsyndromic hearing loss and erythrokeratodermia variabilis. Leveraging the SWISSMODEL server, we established a substantial sequence similarity of 71.4% between Cx30.3 and Cx26, with an identity of approximately 51.1%. Drawing upon this resemblance, we extrapolated the 3D structure of Cx30.3 through homology modeling, guided by the crystal structure of Cx26 (PDB code: 2zw3). Remarkably, a comparative analysis of their structures revealed that the novel mutated residue Glu204 in Cx30.3 corresponds to Glu209 in Cx26, both of which are strategically positioned within the TM4 segment. Within the wild-type Cx30.3 protein, Glu204 participates in 3 distinct hydrogen bonding interactions with Arg22, Ser21 (TM1), and Leu200 (TM3), all of which are confined within a proximity of 5 Å. Additionally, a robust electrostatic interaction connects the acidic Glu204 and the basic Arg22. Collectively, these interactions confer stability to the functional conformation of Cx30.3. However, upon substitution with the smaller, hydrophobic alanine, these stabilizing forces are perturbed, resulting in a consequential impact on the conformational landscape of Cx30.3.

The *GJB4* mutation ( *GJB4*c. 611A>C: p. Glu204Ala) has been categorized as a high-pathogenicity (highPAF) variant, and our study unequivocally demonstrated structural perturbations within Cx30.3. Notably, connexins can form either homomeric or heteromeric gap junction channels, as exemplified by combinations such as Cx26/Cx30, Cx26/Cx32, Cx43/Cx37, Cx43/Cx40, and Cx43/Cx45 [ 12, 29, 30]. However, the precise role of GJB4 mutations in orchestrating hearing impairment within this family context remains enigmatic. Impressively, our findings reveal two significant findings: (1) Both GJB2 and GJB4 mutations are present across all affected individuals. (2) The mutated residue Lys22 in GJB2 resides close to Glu209 and is separated by a mere 5 Å, aligning with the corresponding Glu204 residue in GJB4. Building upon this observation, we postulate that GJB2 and GJB4 might interact synergistically, forming a heteromeric channel (Cx26/Cx30.3) within affected individuals. Consequently, both the Cx26/Cx30.3 heteromeric channel and the Cx26/Cx26 homomeric channel have emerged as pivotal contributors to hearing loss. In this context, the structural disruptions resulted from the coexistence of GJB2 and GJB4 mutations undermine the integrity of the Cx26/Cx30.3 heteromeric gap junction channel, culminating in hearing loss. However, the solitary GJB4 mutation has also been detected within an unaffected population. Conceivably, structural alterations within the heteromeric channel might not extend to the Cx26/Cx26 homomeric channel, thereby preserving its functional integrity and mitigating the risk of hearing loss. Nevertheless, further comprehensive investigations are warranted to substantiate this hypothesis.

In conclusion, our investigation revealed a novel *GJB2* mutation in a Chinese family with deafness via advanced NGS methodology. Leveraging homology modeling and molecular dynamics simulations, we propose that this *GJB2* mutation potentially disrupts the functional conformation of the connexin channel, thereby contributing to the onset of hearing loss. Additionally, while the presence of a *GJB4* mutation introduces the intriguing possibility of being involved in deafness, the intricate relationship between these mutations and their collective impact on hearing impairment demands further exploration in our forthcoming research endeavors.
